# Role of *Aders* and *OXA23* Genes among Imipenem Resistant *Acinetobacter baumannii *Isolates from Two Hospitals of Tehran, Iran

**Published:** 2016

**Authors:** Helia Ostad Asadolah-Malayeri, Mojdeh Hakemi-Vala, Kamibiz Davari

**Affiliations:** 1 *Dept. of Microbiology, Faculty of Basic Sciences, Sanandaj branch,Islamic Azad University, Sanandaj, Iran*; 2 *Dept. of Microbiology, Medical School, Shahid Beheshti University of Medical Sciences Tehran, Iran*

**Keywords:** *Acinetobacter baumannii*, Antibacterial drug resistance, Efflux pump genes, Carbapenemase, OXA-23

## Abstract

**Background:**

This study aimed to evaluate the role of efflux pump regulator and *OXA-23* genes in imipenem resistance *Acinetobacter baumannii* strains isolated from hospitalized patients in Tehran, Iran.

**Methods:**

This study was conducted on 60 *A. baumannii* isolates collected from patients admitted to the Shahid Motahari and Taleghani Hospitals in Tehran during 2013-14. Antibiotic susceptibility tests (AST) and minimal inhibitory concentration (MIC) was determined by broth micro dilution methods according to CLSI 2014 guidelines. The frequency of efflux pump *adeRS *and* OXA-23 *genes were detected by PCR and further sequencing.

**Results:**

The resistance of *A. baumannii* isolates to tested antibiotics was 100% to cefotaxime, ceftazidime, ceftriaxone, ciprofloxacin, cefepime, piperacillin, meropenem, co-trimoxazole and piperacillin/tazobactam, 97% to imipenem, 94% to gentamicin, 83% to amikacin, 76% to tetracycline, and 0.0% to colistin. The MIC of 58 (96.6%) strains to imipenem was highly decreased in the presence of efflux pump inhibitor (PaβN), by 4 to 64 folds. The *adeR and adeS *genes were detected in 36 (60%) and 59 (98.3%), respectively and the frequency of OXA-23 gene was 57 (95%) of isolates.

**Conclusion:**

Existence of *adeRS and OXA-23 *genes in more than 50% of A.* baumannii *isolates in this study shows the presumptive role of efflux pump in simultaneous of carbapenemase production. Therefore, using new strategies are required in order to stop the vertical or horizontal exchanges mentioned genes from the resistant *A. baumannii* isolates to sensitive strains.

## Introduction

*Acinetobacter baumannii (A. baumannii)* has emerged as a highly problematic hospital-associated pathogen and can be an opportunistic pathogen in humans and affecting people with compromised immune systems (1, 2). This ability is related to their long-term survival in the hospital environment, which increases their opportunities for transmission between patients, either via human reservoirs or via insentient materials. Such infections are often extremely difficult for the clinician to treat because of their widespread resistance to the most common antibiotics (multi drug resistant; MDR) (2). Carbapenems, are the last antibiotics in the most nosocomial infections treatment especially in resistant bacteria (3).

Carbapenem resistance in *Acinetobacter* spp. may be mediated by one or sum of further mechanisms such as enzyme production (carbapenemase, oxacillinase), loss of outer membrane protein (OMP), alteration of penicillin-binding protein (PBPs) and specific drug efflux pumps (4).

 Among them, carbapenemases belongs to types A, B and D of Ambler classification (4). Identification of carbapenemase types in *Enterobacteriaceae *and *Pseudomonas *spp. using a phenotypic test to have the common property of hydrolyzing, at least partially, imipenem or meropenem together with other penicillin or cephalosporin antibiotics (4). 

Carbapenemases (type D; OXA enzymes) have emerged globally as the main mechanism responsible for this resistance, although metallo-beta-lactamases (type B) are locally prevalent, especially in East Asia. The OXA carbapenemases of *Acinetobacter *spp. are divided into four phylogenetic subgroups: OXA-23-like; OXA-24-like; OXA-51-like; and OXA-58 (5). 

There are five families of efflux pumps, including the multidrug and toxic compound extrusion (MATE) family, the resistance–nodulation–cell division (RND) family, the adenosine triphosphate (ATP)-binding cassette (ABC) family, the major facilitator superfamily (MFS) and the small multidrug resistance (SMR) family, that are associated with multidrug resistance in bacteria. Amongst them, the RND systems are the most prevalent in MDR *A. baumannii* (6). 

The major clinically relevant efflux systems found in *A. baumannii*, such as AdeABC, AdeIJK and AdeFGH, belong to the (RND) family. AdeABC efflux pump is the first characterized RND system in *A. baumannii*. Overexpression of the AdeABC and AdeIJK efflux pumps has been shown to pump out aminoglycosides, beta-lactam, fluoroquinolone, tetracycline, macrolide, chloramphenicol, trimethoprim, erythromycin, lincosamides, tigecycline (TGC) and also safranin, pyronine, and sodium dodecyl sulfate (7,8). 

The expression of AdeABC efflux pump is tightly regulated by the two-component system, which contains a sensor kinase (SK) AdeS and a response regulator (RR) AdeR, encoded by the adeRS operon transcribed in the opposite direction of AdeABC operon (7). 

In this study, we assessed the simultaneous role of the AdeRS genes and OXA-23 carbapenemase among carbapenem resistance *A. baumannii *isolates from nosocomial infections by the phenotypic and molecular tests.

## Materials and Methods

**Bacterial isolates**

 Sixty no repetitive clinical isolates of carbapenem resistant *A. baumannii* were obtained from 240 clinical samples of two hospitals (Motahari and Taleghani hospitals) in Tehran, Iran during 2013-14.

To ensure the accuracy of genera and species of bacteria, all gram negative nonfermenter bacteria were identified by standard microbial tests(including gram staining, oxidase test, culture on MacConkey agar and TSI (triple sugar Iron agar) and all were confirmed by using Microgen kit (TM Microgen, UK) according to the manufacturer’s protocol.

**Antimicrobial susceptibility testing (AST)**

Antibiotic susceptibility was tested by disk diffusion on Mueller-Hinton agar (Merck, Germany) according to CLSI 2014 guidelines (all antibiotic disks were purchased from Mast Group Ltd, UK.). *E. coli* ATCC25922 and *K.pneunominia* ATCC700603 were evaluated, simultaneously.

**Minimum Inhibitory Concentration (MIC)**

By broth microdilution method, strains, which were resistant to imipenem during the disk diffusion test, were re-checked and their MIC were determined based on the CLSI 2014 (9). The *E. coli* ATCC 25922 was used as a quality control strain, simultaneously. Imipenem powder was purchased from Jaberrebne Hayyan co, Iran. Antibiotic preparation was done based on the manufacturer’s recommendations. Antibiotic concentrations were prepared in the range of 256 µg/ml-0.25µg/ml based on CLSI 2014. Bacterial suspension was prepared equal to 0.5 McFarland’s standard turbidity. Antibiotic serial dilution was prepared in a 96-well microtiter plate (Extra Gene-Company-product No.EL-1190-FCS) contained a total volume of 100 µl of each antibiotic dilution and 90 µl of Mueller-Hinton medium with the 10 µl of diluted bacterial inoculums and microplates were incubated at 37°C for 18 to 24 h. The lowest concentration of antibiotic without visible bacterial growth was defined as the MIC (9).

**Treatment of the Efflux Pump Inhibitor**

As described under microdilution method, changes in susceptibility to imipenem in the presence of 100 µg/ml of the Para amino beta naphtyl amide( PAβN) as an inhibitor was tested. Following the addition of imipenem and the bacterial cell inoculum, 2 µl of the 5-mg/ml stock of either PAβN was added up to 100 µl volume in the microplate . The rest of the procedures were carried out as described above (10). The growth of bacterial suspension in the presence of 100 µl of PAβN was detected for accreditation of the test.

**DNA preparation**

The DNA was extracted by boiling method (11). 

**PCR Procedures**

The presence of two regulatory (adeS and adeR) genes of the AdeABC system, and one of class D carbapenemases (OXA-23) gene were detected by PCR amplification consequently electrophoresis and further staining by ethidium bromide under UV irradiation. The PCR programs, the sequence of the used primers and the product size were shown in Table 1 and 2, respectively. 

**Table1:** The PCR programs were used in this study

Factor	Time					Temperature (ºC)					
Gene	AdeS		AdeR	OXA-23			AdeS		AdeR	OXA-23	
Steps											
Initial denaturation	5min		5min	5min			94°C		94ºC	94°C	
Denaturation.	1min		1min	25sec			94ºC		94ºC	94ºC	
Annealing	1min		1min	40sec			57°C		57°C	56°C	
Extension	1 min		1 min	50sec			72ºC		72ºC	72ºC	
Final extension	7min		7min	6min			72°C		72°C	72°C	
Cycles					30			30			30

**Table 2:** The primer sequences and the products’ size in this study

Primer sequences	Genes	Size	Source of references	Company
**5′ - GATCGGATTGGAGAACCAGA-3′** **5′ -ATTTCTGACCGCATTTCCAT-3′**	OXA-23-F OXA-23-R	501	13	Bioneer/Korea
**5′-ACTACGATATTGGCGACATT-3 5′-GCGTCAGATTAAGCAAGATT-3′**	AdeR-F AdeR-R	447	12	Bioneer/Korea
**5′-TTGGTTAGCCACTGTTATCT-3′** **5′-AGTGGACGTTAGGTCAAGTT-3′**	AdeS-F AdeS-R	544	12	Bioneer/Korea

**Sequencing**

Finally, all PCR products were sequenced by Bioneer Co, Korea) via Takapuzist company (Tehran, Iran). Sequence analysis was done by Chromas 1.45 software and BLAST in NCBI.

**Statistical analysis**

This research was a descriptive study. MINITAB16 software was used for the statistical analyses. The *P* value and confidence intervals were less than 0.05 and 95%, respectively.

## Results

 Overall, 240 different clinical samples were detected from patients referred to Taleghani and Motahari burn hospitals of Tehran, Iran during 2013-14. Continuously, 60 *Acinetobacter* spp. confirmed as *baumannii* were selected randomly. Of included patients, 21 were female and 39 were male. The bacterial isolation based on the hospital wards were 34 were from burn ward, 19 from ICU, 2 of NICU, 2 of nephrology, 1of cardiology, transplant and emergency wards, respectively. In addition, the most frequent isolates were from wound (28), trachea (12), blood (7), sputum (6), stomach and acite fluid (4 each), catheter, CSF, thorax (1), respectively.

**Bacterial identification**

**Fig. 1:**
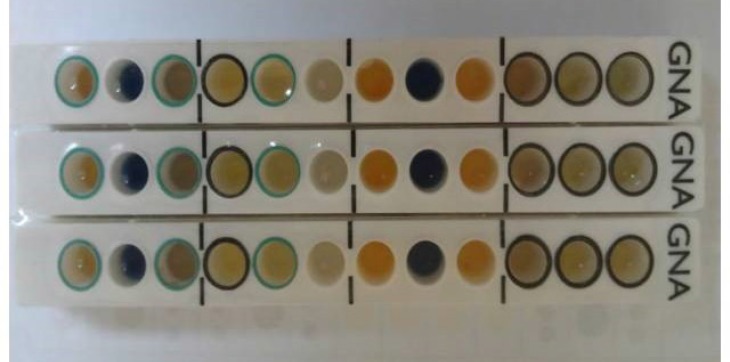
Schema of microgen kit

After Gram staining, oxidase tests, culture on agar MacConkey agar and TSI media and motility. All oxidase-negative, non-fermenter bacteria, non-motile gram-negative bacilli were considered as *Acinetobacter* spp. Further confirmation was done by microgen kit and the results were read on Color chart based on the manufacturer’s instruction. Based on the software analysis all *Acinetobacter* spp. were identified as *baumannii *(Figure1).

**Antibiotic profiles of** ***A.baumannii*** **isolates**

**Fig. 2:**
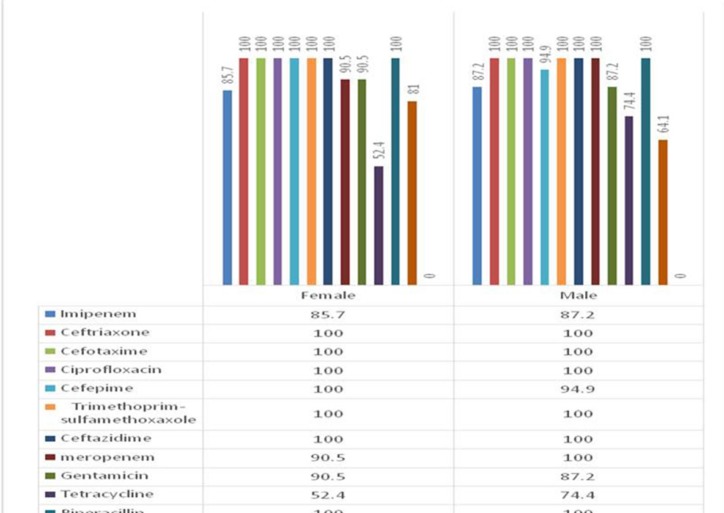
The frequency of antibiotic resistant based on the gender

For all 60 *A. baumannii* isolates, AST was performed and based on the results, 100% of isolates were resistant to ceftriaxone, cefotaxim, ciprofloxacin, trimetoprim-sulfametoxazole, ceftazidim, piperacilin and piperacilin-tazobactam, 96.6% to meropenem and cefepime, 86.6% to imipenem, 88.3% to gentamycin,70% to amikacin and 66.6% to tetracycline. All strains (100%) were sensitive to colistin. All isolates with resistant to more than 3 classes of antibiotics were detected as multi drug resistant (MDR). The frequency of antibiotic resistant based on the gender was shown in the Fig. 2.

**MIC of the imipenem and in accompany of PAβN by microdilution method**

The MIC of imipenem among *A. baumannii* resistant isolates are shown in Table 3. 

**Table 3:** MIC of *A. baumannii* isolates in this study in comparison to CLSI criteria

MIC Interpretive Criteria(μg/ml)			
Species	Resistant	Intermediate	Susceptible
***Acinetobacter baumannii*** **/CLSI 2014**	≥16 μg/ml	8μg/ml	4 μg/ml ≥
***Acinetobacter baumannii/Clinical isolates in this study***	100%	0%	0%

**PCR procedure and sequencing**

**Fig. 3:**
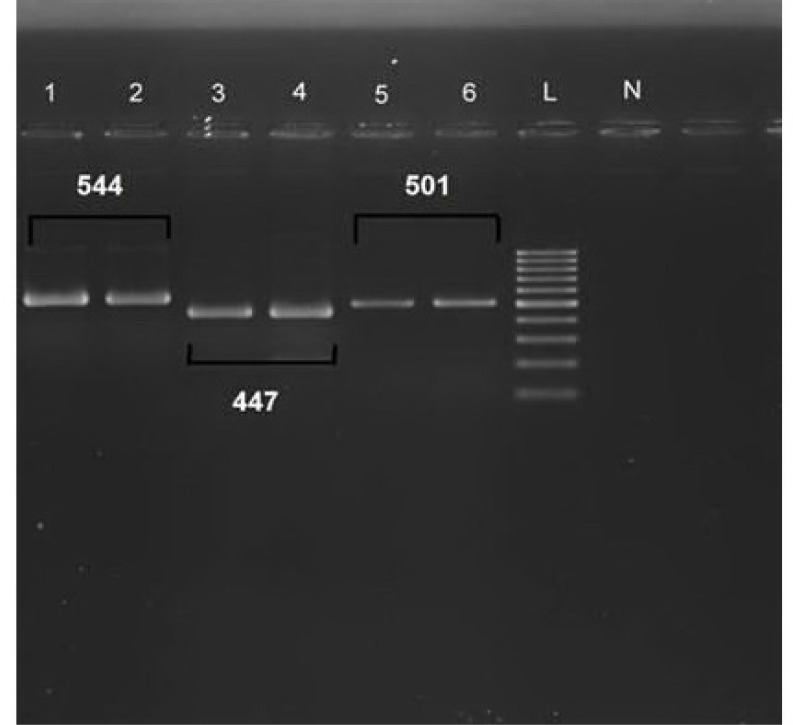
The PCR product size of adeS (544bp), adeR (447bp) and OXA-23(501bp) genes in this study. Lanes1, 2: positive (control strain and a clinical sample) for AdeS gene, lanes 3, 4: positive (control strain and a clinical sample) for AdeR gene, lanes 5, 6: positive (control strain and a clinical sample) gene OXA-23.A 100bp plus-Sina Gene ladder was used in lane L for the comparison

*adeR,* *adeS genes *were detected in 36/60 (60%) and 59/60 (98.3%) respectively, also 57/60(95%) of isolates were positive for existence of OXA-23 gene (Figure 3). Later, confirmation of all PCR products was done after comparison to the databank. Gene submission was done, which is in process.

## Discussion

Existence of 100% resistant to 7 of 14 selected antibiotics by AST showed multidrug resistant among *A. baumannii* isolates in this study. In addition, by microdilution method 100% of isolates were resistant to imipenem. In addition, in this study the PAβN (an inhibitory compound) effect on the imipenem MIC and decrease it during microdilution method. The effect of efflux inhibitory materials such as PAβN was examined in some other studies too. By adding of 100 µg/mL of PAβN it greatly reduced the MIC of ciprofloxacin from 2 to 8 folds (10). Adding of different concentration of PAβN could reduce the MIC of various antibiotics. Outer membrane protein changes and efflux pump expression together may confer resistance to ertapenem in *Enterobacter cloacae*. Similarly, the imipenem susceptibility of most isolates was increased in the presence of PaβN, mainly from 4 to 64 folds (12).

The prevalence of AdeS, AdeR, and OXA-23 genes among the *A. baumannii* isolates of this study was 98.3%, 60% and 95%, respectively. 

In China, the relationship between resistance to imipenem and efflux pumps AdeABC, AdeM, AdeDE, were evaluated among the clinical *A. baumannii* isolates. 80% of *A. baumannii* strains, which were resistant to imipenem, harbored AdeB, AdeR, Ades and AdeJ genes (13). In accordance to this study, the frequency of AdeRS gene among imipenem resistant *A. baumannii* strains was investigated and AdeS and AdeR genes were observed in 98.3% and 60% of strains, respectively. 

The frequencies of the blaIMP2, blaGES1, blaVIM2, blaOXA-23, blaOXA-51genes were evaluated among *A. baumannii* strains collected from seven hospitals in Tehran: and the frequency of blaOXA-23 and blaOXA-51 was 94% and 84%, respectively (14). In the present study, although the frequency of genes blaIMP2, blaGES1 and blaVIM2 was not examined, but the frequency of OXA-23 gene was 95%, similarly. This similarity despite the time intervals (about 2 yr difference) between two studies, is another confirmation for the results of the recent study.

The role of RND efflux pumps in *A. baumannii *resistance to biocides was investigated by RT-PCR (15) and found that increased expression of AdeB and AdeJ genes causes increased resistant to biocides and the frequency of AdeS, AdeR, AdeB genes were observed in 55% of strains (15). In the present study, although gene expression by RT-PCR was not examined, but the frequency of AdeR and AdeS genes were assessed by PCR and they were seen in 60% and 98.3% of strains, respectively. High frequency rate of mentioned genes in this study may be related to increased rate of antibiotic consumption or drug resistant recently. 

In Korea, researchers evaluated the role of efflux pump AdeABC and OXA-23 in *A. baumannii* strains carrying OXA-66 by using PFGE and real time PCR. According to the results, with PFGE technique and digestion by the enzyme SmaI, all isolates had the same restriction pattern. Isolates’ MIC of meropenem were found ≥ 32 mg/L. By real time PCR technique, the expression level of AdeB gene in 6 isolates of *A. baumannii* was 10 to 40 times greater than the strains susceptible to imipenem and all isolates had blaOXA-23 gene. Finally, they have announced that increased expression of OXA-23 gene and AdeABC efflux pump may have an important role in the emergence of the carbapenems resistance *A. baumannii* isolates (16). 

In accordance to this study, 100% of isolates were resistant to imipenem based on detected MIC by microdilution method and existence of the OXA-23 gene was observed in 95% of isolates. However, the existence of AdeABC was not evaluated in this study but in our other study, existence of AdeAB efflux genes and AdeC gene among *A. baumannii *isolates were 100% and 85%, respectively (17).

In a Brazilian study, antimicrobial susceptibility was tested by the disk diffusion method and then, samples resistant to imipenem were evaluated by using PCR for the presence of blaOXA-23 gene. Based on the results, 95.4% of the samples had blaOXA-23 gene and the amount of resistance to imipenem and meropenem was 71.4% and 69.7%, respectively (18). Similarly, in this study, sensitivity to antibiotics was tested by disk diffusion method and 86.6% and 96.6% of the strains were resistant to imipenem and meropenem, respectively. In addition, in 95% of strains, OXA-23 gene was found. 

In Saudi Arabia, 55 isolates of *A. baumannii* isolates were collected. These bacteria were resistant to aztereonam, cefepim, ceftazidim and imipenem. Then, E-test for MIC was laid. The presence of ß-lactamase genes were evaluated by PCR. The genes blaOXA-23, blaPER, blaGES, and blaOXA-24 were observed in 60%, 49.1%, 34.5%, and 3.6% of samples.

 The genes SHV, CTX-M, VEB, KPC, OXA-58 and metalo-beta-lactamases were not observed. In addition, the samples, which showed high resistance to carbapenems had blaOXA-23 and blaOXA-24 genes (19). In the present study, MIC was determined using microdilution method for imipenem and the samples were undergone PCR to evaluate the presence of genes OXA-23, AdeR, and AdeS. According to CLSI 2014, by using MIC test, resistance to imipenem was shown in 100% of samples. The comparison of two studies can clearly show the direct link of MIC and the presence of OXA-23 gene. On the other hand, the higher of the percentage of OXA-23 existence, shows the higher of the percentages of resistant to imipenem.

In Taiwan, the molecular epidemiology and antimicrobial resistance in Multi Drug Resistance (MDR) *Acinetobacter* spp were evaluated in 5 hospitals in Taiwan. By using PCR technique, the presence of following genes: intI1, bla_IPM_, bla_VIM_, bla_OXA-51_, bla_OXA-23_, bla_OXA-58_, bla_OXA-24_, bla_ompC_, adeB, adeJ, adeM were evaluated. According to the PCR results, the prevalence of the tested genes was 91% for intI, 57% for blaOXA-23 and 100% for bla_ompC_, adeB, adeJ and adeM. Determining the resistance genes in *A. baumannii* isolates is necessary to reduce emergence of resistance bacteria (20). In the present study, samples were evaluated by PCR in terms of the presence of the genes OXA-23, AdeR, and AdeS and MIC was determined using microdilution method for imipenem. OXA-23 gene was present in 95% of samples and according to disk diffusion method, 100% of isolates were imipenem resistant. By comparing these two surveys, existence of OXA-23 gene is related to high carbapenem resistance.

In France, RND efflux pumps were evaluated in 14 MDR. *A. baumannii* isolates and the role of the regulating AdeRS genes were studied in terms of mutation by Multi Locus Sequence Typing (MLST) and Pulsed-Field Gel Electrophoresis (PFGE) methods. Functional mutation in protected areas AdeRS was seen in all the strains in which over expression of AdeABC was present. Mutations were found in two hot spots of AdeS (near histidine 149) and another in the DNA binding domain of AdeR. Also they showed, high incidence of over expressed efflux pump AdeABC among MDR *A. baumannii *isolates as a result of a variety of individual mutations is in the corresponding of this two-component regulatory system (21). In the present study, although MLST and PFGE techniques were not used, but the existence and the frequency of AdeRS genes were examined by PCR and further sequencing and the presence of AdeRS genes was seen in more than half of the bacterial isolates.

## Conclusion

Existence of *adeR, adeS (*60%, 98.3% respectively) and* OXA-23 (*95%*) *genes in more than half of A.* baumannii *isolates in this study shows the presumptive role of both efflux pump in simultaneous of carbapenemase production during antibiotic resistance of *A. baumannii* isolates. So, using new strategies are required in order to stop the vertical or horizontal exchanges of the efflux pump and OXA-23 genes from the resistant *A. baumannii* isolates to sensitive strains. Also by doing AST before any prescription can decrease the emergence of resistant *A. baumannii *isolates.

## Authors’ Contributions

The core idea of this work came from Mojdeh Hakemi- Vala. Dr. Kambiz Davari was another advisor in this project. Helia Ostadasadolah-Malayeri collected and cultured the clinical samples. All the remained phenotypic, molecular tests and manuscript draft was done by her. Final article revision and data analysis was done by Mojdeh Hakemi- Vala.
